# Exercise activates the PI3K-AKT signal pathway by decreasing the expression of 5α-reductase type 1 in PCOS rats

**DOI:** 10.1038/s41598-018-26210-0

**Published:** 2018-05-22

**Authors:** Chuyan Wu, Feng Jiang, Ke Wei, Zhongli Jiang

**Affiliations:** 1 Department of Rehabilitation Medicine, the First Affiliated Hospital of Nanjing Medical University, Nanjing, China; 2 Neonatal Department, Obstetrics and Gynecology Hospital of Fudan University, Shanghai, China; 3 Medical Service Section, the First Affiliated Hospital of Nanjing Medical University, Nanjing, China

## Abstract

Hyperandrogenism and hyperinsulinemia are main clinical endocrine features of PCOS. Exercise can adjust the androgen level, as well as increase the sensitivity of insulin by activating PI3K-Akt insulin signaling pathways. 5αR1 has certain effects on insulin resistance and can synthesize dihydrotestosterone by metabolizing testosterone. So 5αR1 may be the target of androgen and insulin for exercise-induced regulation. To investigate the role of 5αR1 in the PI3K-Akt signaling pathway in skeletal muscle of PCOS rats activated by exercise, fifty-four female rats were randomly divided into the PCOS group (n = 42) and the control group(n = 12). After injection of testosterone propionate for 28 days, the remaining 36 rats in the PCOS group were randomly assigned to six groups: the sedentary group (PS, n = 6), sedentary and 5αRI (5α-reductase inhibitor) group (PS + RI, n = 6), sedentary and 5αR2I (5α-reductase type 2 selective inhibitor) group (PS + R2I, n = 6), exercise group (PE, n = 6), exercise and 5αRI group (PE + RI, n = 6), and exercise and 5αR2I group (PE + R2I, n = 6). The rats undergoing exercise were trained to swim for 14 days. Finasteride (5α-reductase type 2 selective inhibitor) and dutasteride (5α-reductase inhibitor) were administered once daily and were dosed based on weight. At the end, the expression of 5αR1 proteins, the phosphorylation level of PI3K and AKT, were determined by Western blot. The PCOS non-exercise group and the PE + RI group displayed significantly lower phosphorylation of Akt, PI3K p85 and GLUT4 expression, while in the PE + R2I group, the level of Akt phosphorylation and PI3K p85 expression was significantly higher than that of the PCOS non-exercise group and the PE + RI group. In summary, our study demonstrated that exercise can activate the PI3K/AKT signal pathway of PCOS rats by decreasing the expression of 5αR1.

## Introduction

Polycystic ovary syndrome (PCOS) is a clinically heterogeneous syndrome characterized by anovulation, clinical or biological hyperandrogenism and abnormalities of the ovary^1^. It is one of the most common endocrine diseases in women of reproductive age^2^. The main clinical endocrine features of this syndrome are hyperandrogenism and hyperinsulinemia^1,3^. Currently, a variety of medications (such as ovulation drugs, anti-androgen drugs, and insulin sensitizer) have been used for the treatment of PCOS; however, the majority of these drugs are directed only to one specific feature of this syndrome and there remains some difficulty in decreasing the level of androgen while improving insulin sensitivity. Exercise can simultaneously improve the two major endocrine characteristics of PCOS.

Exercise therapy can increase insulin sensitivity and decrease the level of androgen, and the effects of exercise therapy in PCOS patients are becoming increasingly important^4–6^. Obese PCOS patients can restore normal menstrual cycle and pregnancy after diet and exercise intervention, and if exercise therapy is performed prior to receiving assisted reproductive technology, the pregnancy rate can be significantly increased^7,8^. Previously, we have successfully generated an obesity rat model of PCOS. In these experiments, exercise and dietary intervention were used in the obese PCOS rats to demonstrate that exercise training improved insulin sensitivity and decreased androgen levels^5^. The signal transduction pathway of insulin and glucose metabolism has been extensively studied. In recent years, many scholars have paid close attention to the relationship between the phosphatidylinositol 3-kinase (PI3K)/protein kinase B (Akt) signaling pathway and polycystic ovary syndrome^9^. The PI3K/Akt signaling pathway plays an important role in regulating metabolism. When the insulin receptors are activated, they phosphorylate the insulin receptor substrate (IRS), which then binds to the PI3K protein, activating PKC, Akt and the downstream signal molecule AS160. This prompts translocation of glucose transporter type 4(GLUT4) and allows muscle cells to absorb more glucose, hence regulating glucose metabolism. Thus, the alterations in the PI3K-Akt signaling pathway are closely related to insulin resistance (IR), diabetes mellitus, and metabolic diseases. Importantly for these studies, this signaling pathway can be used by contracting muscles^7^.

Androgen is a protein and an anabolic hormone that plays a role in promoting the use of energy reserves in muscle cells and participates in the signal transduction pathway of glucose metabolism in skeletal muscle cells^10,11^. In skeletal muscle cultures from normal SD rats, increasing exogenous androgen concentration could modulate the activation of PKC and Akt. The activation of Akt could be inhibited by androgen related inhibitors^12^. Thus, PKC/Akt/GLUT-4 is likely to be a common pathway for insulin and androgen signaling pathways in glucose metabolism. In 2014, the latest international research results showed that androgen did not interact with insulin in regulating the level of glucose metabolism of skeletal muscle in patients with PCOS. This was shown through direct glucose uptake experiments^13^. This raises a contradiction and allows us to speculate that there is a genetic or protein expression deficit in PCOS patients and that there is lack of some androgen triggering mechanism of glucose metabolism.

**Figure 1:**
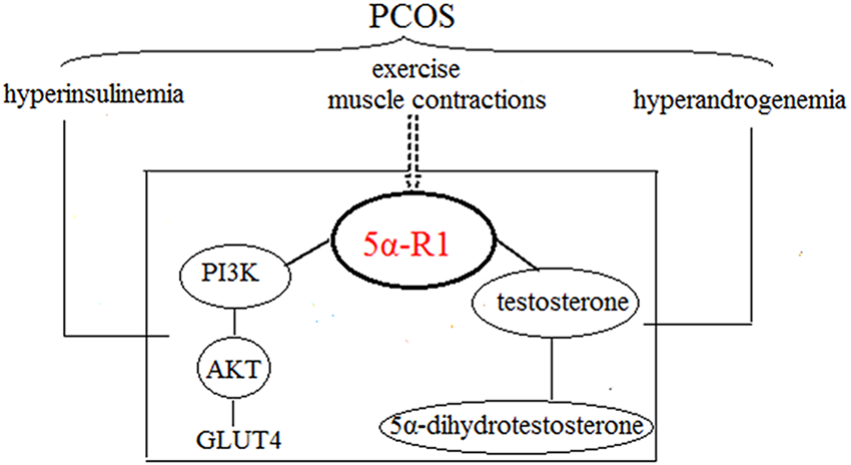
Schematic diagram of the research hypothesis. Hyperandrogenism and hyperinsulinemia are main clinical endocrine features of PCOS. Exercise can adjust the androgen level, as well as increase the sensitivity of insulin by activating PI3K-Akt insulin signaling pathways. 5αR1 has certain effects on insulin resistance and can synthesize dihydrotestosterone by metabolizing testosterone. 5αR1 may be the target of androgen and insulin for exercise-induced regulation.

An important metabolic enzyme in the body that regulates androgens is known as the 5α-reductase enzyme (5αR). Two types of isoenzymes named 5α-reductase type 1(5αR1) and 5α-reductase type 2(5αR2) are encoded by the 5α-reductase gene type 1(SRD5A1) and the 5α-reductase gene type 2(SRD5A2), respectively. In 2009, Boda confirmed that the presence of polycystic ovary syndrome in women was due to an abnormal expression of 5αR^14^. A 2014 population study confirmed that 5αR blockers increased insulin resistance and were more likely to be after 5αR1 was inhibited^15^. Population studies confirmed that 5αR1 has certain effects on insulin resistance. Exercise can adjust the androgen level, as well as increase the sensitivity of insulin. Thus, 5αR1 may be the target of androgen and insulin for exercise-induced regulation (Fig. 1).

In the present study, we activated PI3K-Akt insulin signaling pathways in the skeletal muscle of PCOS rats by exercise. To investigate the role of 5αR1 in the PI3K-Akt signaling pathway in skeletal muscle of PCOS rats activated by exercise, we compared dutasteride, a dual 5αR1 and 5αR2 inhibitor, with finasteride, a 5αR2 selective inhibitor.

## Materials and Methods

### Ethics statement

Fifty-four Wistar female rats (50–70 g, 21 days old) were purchased from the Beijing Vital River Laboratory Animal Technology Co. (Beijing, China). The animals were housed in temperature-controlled rooms (22 °C), with light from 08:00 to20:00 h and dark from 20:00 to 08:00 h and had free access to water. The experimental protocols were approved by the Institutional Animal Care and Use Committees of Nanjing Medical University (permit number: NJMU/IA-CUC_20120210_01) and strictly followed. The experimental protocols were in strict accordance with the Guidelines for the Care and Use of Laboratory Animals (National Research Council of People’s Republic of China, 2010).

### Study procedure

**Figure 2:**
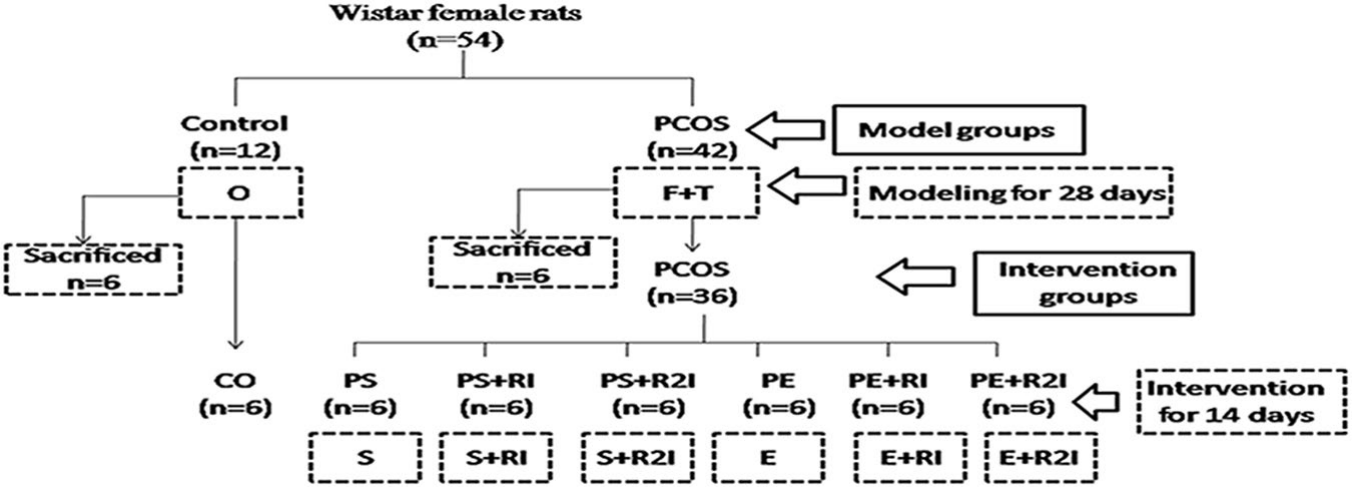
The experimental design. O: ordinary diet; F: high fat diet; T: testosterone propionate; Control: control group; PCOS: PCOS model group; S: no exercise; PS + RI: sedentary with 5 alpha R inhibitor in PCOS rats; PS + R2I: sedentary with 5 alpha R2 inhibitor in PCOS rats; E: exercise; PE + RI: exercise with 5 alpha R inhibitor in PCOS rats; PE + R2I: exercise with 5 alpha R2 inhibitor in PCOS rats.

All rats were randomly divided into two experimental groups (Fig. 2): the PCOS model group (PCOS, n = 42) and the Control group (control, n = 12). The PCOS rat model was generated by injecting testosterone propionate for 28 days and being fed a high-fat diet according to our previous studies. The day after the 28th injection, a total of 12 rats from the PCOS group and the Control group were sacrificed by cervical dislocation. When rats were sacrificed, blood samples were obtained from all the 12 rats and ovaries were dissected from connective tissue to verify the PCOS phenotype^5^. Then six rats were included in each group.

The remaining 36 rats in the PCOS group were randomly assigned to six groups (Fig. 1): the sedentary group (PS, n = 6), sedentary and 5αRI group (PS + RI, n = 6), sedentary and 5αR2I group (PS + R2I, n = 6), exercise group (PE, n = 6), exercise and 5αRI group (PE + RI, n = 6), and exercise and 5αR2I group (PE + R2I, n = 6). All the remaining 42 rats were fed with an ordinary diet for two weeks. The exercised rats in the PE, PE + RI and PE + R2I groups swam simultaneously without a load for 120 min/d, 6 days/week for two weeks in a barrel filled with water maintained at 33–35 °C at a depth of 40–50 cm. Every rat was given enough area to swim freely^6,16^. The cages of non-exercised rats in the PS, PS + RI and PS + R2I groups were placed nearby the barrels to eliminate the influence of exercise noise^6^. Finasteride (5 mg/kg/day)^17^ and dutasteride (1 mg/kg/day)^18^ were administered by gavage in the PE + RI, PE + R2I, PS + RI and PS + R2I groups, once daily. All of the rats were sacrificed between 08:00 and 11:00 AM under fasting conditions 24 h after the end of the last training session with 3% pentobarbital abdominal anesthesia followed by cervical dislocation. Blood and skeletal muscle (mainly composed of gastrocnemius muscle) samples were collected from all animals.

### Measurements of body weight and body length

The body weight and length of rats were measured weekly beginning at 21days of age. The body length was defined as the distance from the nose to the anus of the rats. The Lee index reflects the body fat as a parameter[LI = body weight (g)^1/3^ × 1000/body length (cm)]^5^.

### Oral glucose tolerance test (OGTT)

Prior to sacrificing, all rats were fasted overnight and infused intragastrically with 2 g glucose per kilogram of body weight. Blood samples were collected at 0 min, 30 min, 60 min and 120 min after oral glucose was administered to evaluate fasting blood glucose (FBG), 30 min postprandial blood glucose (PBG30′), 1 h postprandial blood glucose (PBG60′) and 2 h postprandial blood glucose (PBG120′), respectively^19^.

### Serum analysis

Blood samples obtained from all rats fasting for 12 h were centrifuged at 2500 g for 10 min and stored at −80 °C. Fasting serum levels of insulin (FINS) and testosterone (T) were determined using commercial ELISA kits (Jiancheng Bioengineering Institute, Nanjing, P. R. China). Fasting blood glucose (FBG), 30 min postprandial blood glucose (PBG30′), 1 h postprandial blood glucose (PBG60′) and 2 h postprandial blood glucose (PBG120′) were analyzed by GOD-PAP. AUC_glucose_(Area under the curve of blood glucose) = 1/4 × FBG + 1/2 × PBG30′ + 3/4 × PBG60′ + PBG120′^20^. The insulin resistance index is defined as the ratio of HOMA-IR (Homeostasis model assessment)^21^.

### Western blotting analysis

One hundred milligrams of skeletal muscle from the gastrocnemius muscle of the different groups were stored at −80 °C and were homogenized in 10–20 volumes with a buffer containing 1% sodium dodecylsulfate(SDS), 100 mmol/L Tris HCl(pH6.8), 1 mmol/L phenylmethyl sulfonyl fluoride (PMSF), and 0.1 mmol/L β-mer-captoethanol. The homogenate was centrifuged at 12000 × g for 10 min at 4 °C and supernatant A was collected. Then, supernatant A for the protein GLUT4 was centrifuged at 4 °C, 40000 × g/1 h to precipitate the cell membrane debris. The supernatant was centrifuged again at 4 °C, 40000 × g for 10 min, with the proper amount of membrane protein extraction reagent to obtain the supernatant after high-speed mixing using a vortex mixer after 5 seconds. Then, the sample was placed in an ice bath precipitation for 5–10 minutes, which was repeated 3 times to obtain the full extraction of protein. At 4 °C, 40000 × g/15 min, supernatant B for PM GLUT4 was collected for the membrane protein solution. The protein concentration was determined by the BCA protein assay. For each sample, proteins were separated by electrophoresis via 10% sodium dodecylsulfate-polyacrylamide gel electrophoresis (SDS-PAGE). The gel was transferred to a nitrocellulose (NC) membrane (Bio-Rad Instruments, CA, USA) in transfer buffer containing 25 mmol/L Tris, 192 mmol/L glycine, and 20% methanol. The NC membranes were blocked using PBS containing 2% bovine serum albumin (BSA) and 0.05% Tween-20 at 4 °C for 1 h at room temperature and incubated with primary antibody overnight. The following antibodies were used for the supernatant A: anti-Akt antibody, anti-Phospho-Akt(Ser473) antibody, anti-Phospho-Akt (Thr308) antibody, anti-PI3 Kinase p85 antibody(Cell Signaling Technology, Danvers, MA), anti-SRD5A1 antibody, and anti-GLUT4 antibody(Abcam, Cambridge, UK).The next day, membranes were incubated with goat anti-rabbit-HRP secondary antibody(Abcam, Cambridge, UK) at room temperature for 1 h. Photos were acquired using a gel imaging analysis system (Tanon 6600; Tanon shanghai). Quantification analysis of blots was performed with Image-Pro plus 6.0 software. The same procedure was also for the supernatant B to detect the expression of PM GLUT4.The relative amount of target proteins were represented by the protein/β-actin gray-scale ratio and phosphorylation was expressed as the ratio of phosphorylated to total protein.

### Immunohistochemical staining for GLUT4 protein expression

The formalin-fixed paraffin-embedded tissue sections were deparaffinized in xylene, followed by rehydration in decreasing ethanol concentrations at room temperature. Retrieval with high-temperature heating in citrate buffer (10 mM citrate, pH6.0) was performed and the slides were incubated with inhibitor buffer (3% H2O2) and washed with PBS (0.01 mol/L). The slides were treated with goat anti-rat GLUT4 primary antibody (Abcam, Cambridge, UK) overnight at 4 °C. After another washing with PBS, the slides were incubated with rabbit anti-goat secondary antibody polymer-based En Vision-HRP-enzyme conjugate (Abcam, Cambridge, UK) at room temperature. The slides were observed under an optical microscope and 10 non-overlapping high-magnification fields were selected after being stained with 3,3′-diaminobenzidine (DAB). The average optical density values of every field were measured using Image-Pro plus 6.0 software.

### Statistical analyses

Data were expressed as the means ± SD. Statistical evaluations were performed with SPSS software (version 16.0, SPSS, Chicago, IL, USA). All of the data were analyzed by T test or one-way ANOVA, and P < 0.05 was considered significant. When the ANOVA revealed significant differences among the three experimental groups, post hoc analysis was performed using least significant difference (LSD), and statistical difference was set at P < 0.05.

## Results

### Changes in metabolic and endocrine variables in a PCOS model induced by a high-fat diet and testosterone

The metabolic and endocrine variables were measured on the 28^th^ day of the experiment in PCOS and Control groups. The Lee index in the PCOS group was significantly increased in comparison with the Control group. Serum levels of FINS and T were significantly increased in the PCOS group than in the Control group. The levels of FBG and PBG1were significantly higher in the PCOS group than in the Control group. The levels of AUC_glucose_ were significantly increased in the PCOS group compared to the Control group. The value of HOMA in the PCOS group was also significantly higher compared with the Control group (Table 1).

**Table 1:** Changes of metabolic and endocrine variables in PCOS model.

	Control	PCOS	P (PCOS vs. Control)
Lee Index	295.44 ± 5.48	304.11 ± 5.25	0.039
T(nmol/L)	31.58 ± 1.19	38.96 ± 5.18	0.025
DHT(nmol/L)	29.72 ± 2.93	30.69 ± 2.87	0.597
FINS(mU/L)	10.28 ± 2.02	11.35 ± 1.21	0.048
FBG (mmol/L)	2.95 ± 0.33	3.88 ± 0.74	0.021
PBG30′(mmol/L)	7.86 ± 1.50	8.97 ± 1.87	0.315
PBG60′(mmol/L)	7.74 ± 0.90	10.92 ± 1.55	0.003
PBG120′(mmol/L)	4.58 ± 0.93	5.48 ± 1.26	0.208
AUC glucose(h.mmol/L)	13.24 ± 1.56	15.88 ± 1.40	0.016
HOMA-IR	1.35 ± 0.32	1.77 ± 0.31	0.045

### 5αR1expression in skeletal muscle of PCOS rats

**Figure 3:**
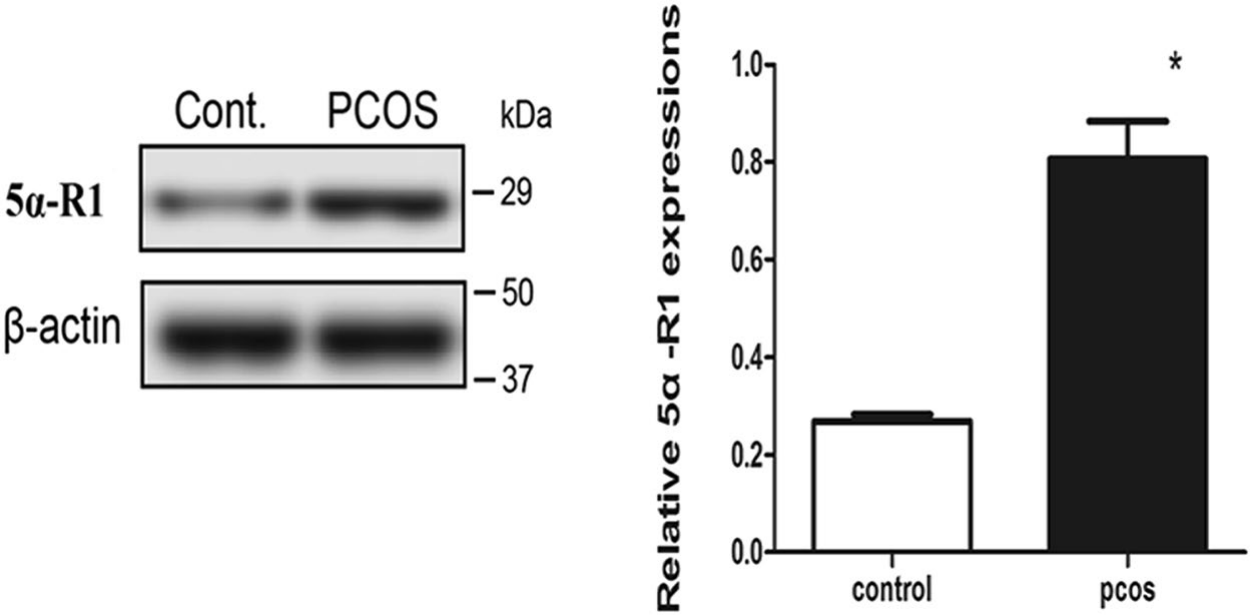
Ratios of 5αR1 to β-actin. *Compared with the Control group, P < 0.05.

Compared with the Control group, the expression of 5αR1 was significantly higher in skeletal muscle of the PCOS group (Fig. 3).

### Changes in metabolic and endocrine variables in response to exercise and 5αR inhibition

The metabolic and endocrine variables were measured at the end of the experiment in the CO, PS, PS + RI, PS + R2I, PE, PE + RI and PE + R2I groups. Compared with the PS group, the levels of FINS were significantly decreased in the PE and PE + R2I groups. The levels of PBG120′ were significantly increased in the PS group compared with the CO group and they were significantly decreased in the PE + R2I group compared to the PS and PS + RI groups. The levels of AUG glucose and HOMA-IR were significantly elevated in the PS, PS + RI, PS + R2I and PE + RI groups compared to the CO group, while the levels of HOMA-IR were significantly reduced in the PE + R2I compared to the PS, PS + RI, PS + R2I and PE + RI groups. No significant differences were found in the levels of FBG, PBG30′ and PBG60′ in all of these groups (Table 2).

**Table 2:** Changes of metabolic and endocrine variables after exercise.

	No-exercise				exercise		
	CO	PS	PS + RI	PS + R2I	PE	PE + RI	PE + R2I
FINS (mU/L)	10.30 ± 0.69	11.45 ± 0.71^a^	11.01 ± 0.68	10.93 ± 0.71	10.13 ± 0.64^b^	11.42 ± 0.87^ae^	9.67 ± 1.23^bcdf^
FBG (mmol/L)	3.12 ± 0.34	3.06 ± 0.38	3.35 ± 0.38	3.60 ± 0.60	2.93 ± 0.41	3.33 ± 0.31	3.05 ± 0.27
PBG30′ (mmol/L)	7.2 ± 1.04	9.22 ± 2.44	8.13 ± 0.61	8.02 ± 1.40	8.47 ± 0.92	8.06 ± 1.13	9.65 ± 2.96
PBG60′ (mmol/L)	6.32 ± 0.34	7.57 ± 0.77	8.03 ± 1.44	7.70 ± 1.02	6.73 ± 0.88	7.92 ± 0.52	7.90 ± 2.13
PBG120′ (mmol/L)	4.20 ± 0.42	5.12 ± 0.74^a^	4.80 ± 0.43	4.37 ± 0.97	4.33 ± 0.44^b^	4.46 ± 0.48	3.68 ± 0.72^bc^
AUC_glucose_ (h.mmol/L)	13.35 ± 0.97	16.04 ± 1.37^a^	15.73 ± 0.81^a^	15.05 ± 1.30^a^	14.35 ± 0.41^b^	15.27 ± 0.81^a^	15.14 ± 3.34
HOMA-IR	1.43 ± 0.14	1.56 ± 0.10^a^	1.64 ± 0.21^a^	1.74 ± 0.22^a^	1.32 ± 0.16^c^	1.70 ± 0.25^ae^	1.31 ± 0.22^bcdf^

Values are means ±SD. ^a^compared with CO group, p < 0.05; ^b^compared with PS group, p < 0.05; ^c^compared with PS + RI group, p < 0.05; ^d^compared with PS + R2I group, p < 0.05; ^f^compared with PE + RI group, p < 0.05.

### 5αR1expression in skeletal muscle in response to exercise and 5αR inhibition

**Figure 4:**
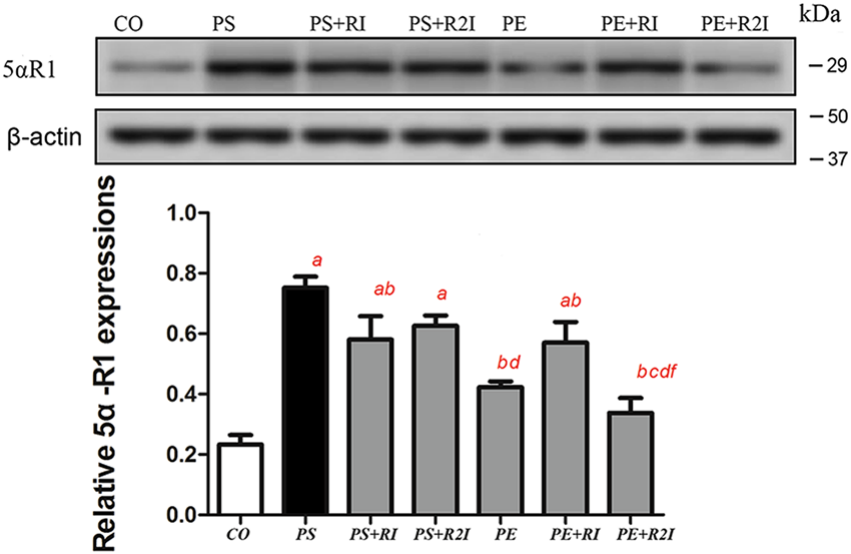
Ratios of 5αR1 to β-actin; response to exercise and 5αRI. ^a^Compared with the CO group, p < 0.05; ^b^compared with the PS group, p < 0.05; ^c^compared with the PS + RI group, p < 0.05; ^d^compared with the PS + R2I group, p < 0.05; ^f^compared with the PE + RI group, p < 0.05.

After 14 days of intervention, the expression of 5αR1 in the PS, PS + RI, PS + R2I and in the PE + RI groups were significantly increased compared with the CO group. Compared with the PS group, the expression of 5αR1 was significantly lower in the PS + RI, PE, PE + RI and PE + R2I groups (P < 0.05). No significant difference was observed between the CO group, the PE group and the PE + R2I group (Fig. 4).

### PI3Kp85 and Akt phosphorylation expression in skeletal muscle in response to exercise and 5αR inhibition

**Figure 5:**
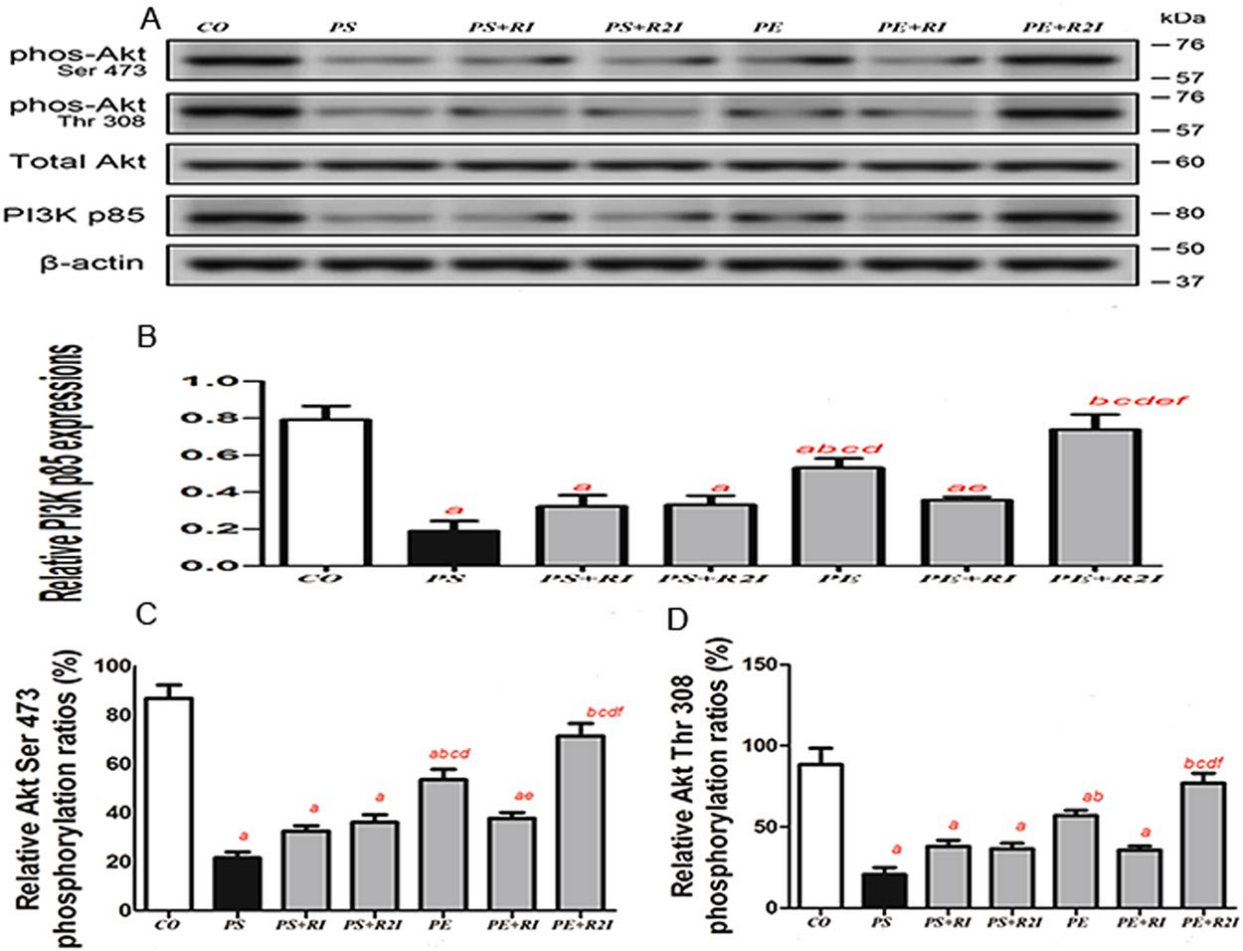
Ratios of PI3Kp85 to β-actin and Akt phosphorylationto total Akt in skeletal muscle in response to exercise and 5αR inhibition. ^a^Compared with the CO group, p < 0.05; ^b^compared with the PS group, p < 0.05; ^c^compared with the PS + RI group, p < 0.05; ^d^compared with the PS + R2I group, p < 0.05; ^f^compared with the PE + RI group, p < 0.05.

Compared with the CO group and the PE + R2I group, the expression of PI3Kp85 was significantly reduced in the PS, PS + RI, PS + R2I, PE and PE + RI groups. Compared with the PE group, the expression of PI3Kp85 was significantly lower in the PS, PS + RI and PS + R2I groups (P < 0.05). There was no significant difference in the expression of PI3Kp85 between the CO group and the PE + R2I groups, as well as between the PS, PS + RI, PS + R2I and PE + R2I groups (Fig. 5).

Compared with the CO group and the PE + R2I group, the relative Akt(Ser 473) phosphorylation ratios were significantly lower in the PS, PS + RI, PS + R2I, PE and PE + RI groups (P < 0.05). Compared with the PE group, the relative Akt(Ser 473) phosphorylation ratios were significantly lower in the PS, PS + RI, PS + R2I and PE + RI groups(P < 0.05). There was no significant difference in the expression of PI3Kp85 between the CO, PE and PE + R2I groups, as well as between the PS, PS + RI,PS + R2I and PE + R2I groups (Fig. 5).

Compared with the CO and the PE + R2I group, the relative Akt(Thr308) phosphorylation ratios were significantly lower in the PS, PS + RI, PS + R2I and PE + RI groups (P < 0.05). Compared with the PE group, the relative Akt(Thr308) phosphorylation ratios were significantly reduced in the PS group (P < 0.05). No significant difference was found in the expression of PI3Kp85 between the CO and PE + R2I groups, as well as between the PS, PS + RI and PE + R2I groups (Fig. 5).

### Effects of exercise and 5αR inhibition on GLUT4 expression

**Figure 6:**
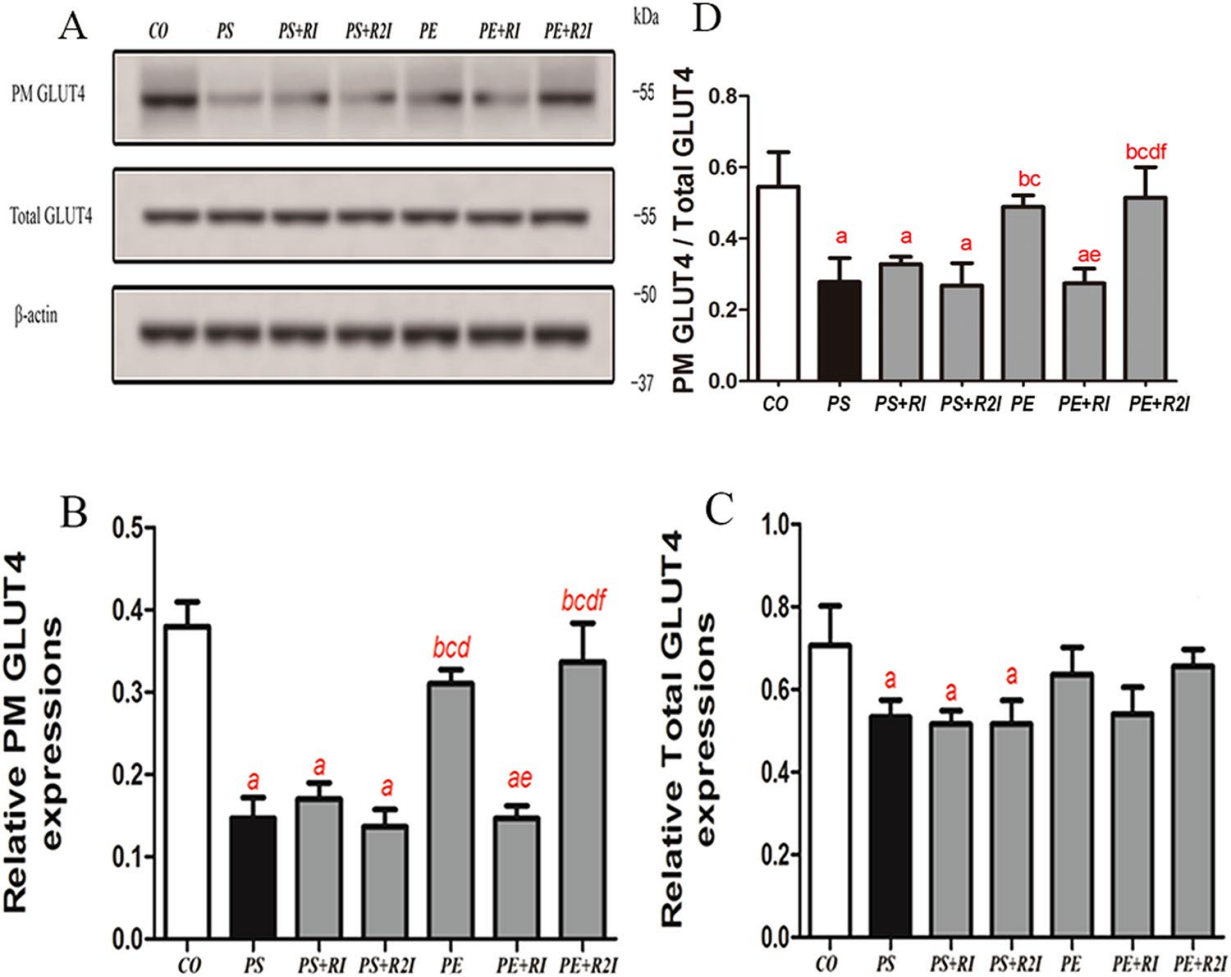
Effect of exercise and 5αRI on GLUT4 expression in skeletal muscle. (**A**) Western blots of PM GLUT4 and total GLUT4. (**B**) Ratios of PM GLUT4 to β-actin. (**C**) Ratios of total GLUT4 to β-actin. (**D**) Ratios of PM GLUT4 to total GLUT4. ^a^Compared with the CO group, p < 0.05; ^b^compared with the PS group, p < 0.05; ^c^compared with the PS + RI group, p < 0.05; ^d^compared with the PS + R2I group, p < 0.05; ^f^compared with the PE + RI group, p < 0.05.

Compared with the CO, PE and PE + R2I groups, the expression of PM GLUT4 was significantly lower in the PS, PS + RI, PS + R2I and PE + RI groups (P < 0.05). Compared with the CO, the expression of total GLUT4 was significantly reduced in the PS, PS + RI and PS + R2I groups (P < 0.05). There was no significant difference in the expression of PM GLUT4 and total GLUT4 between the CO, PE and PE + R2I groups, as well as between the PS, PS + RI,PS + R2I and PE + R2I groups. The ratio of PM GLUT4 to total GLUT4 also tend to be significantly lower in the PS, PS + RI, PS + R2I and PE + RI groups (P < 0.05) (Fig. 6).

### GLUT4 immunocytochemistry

**Figure 7:**
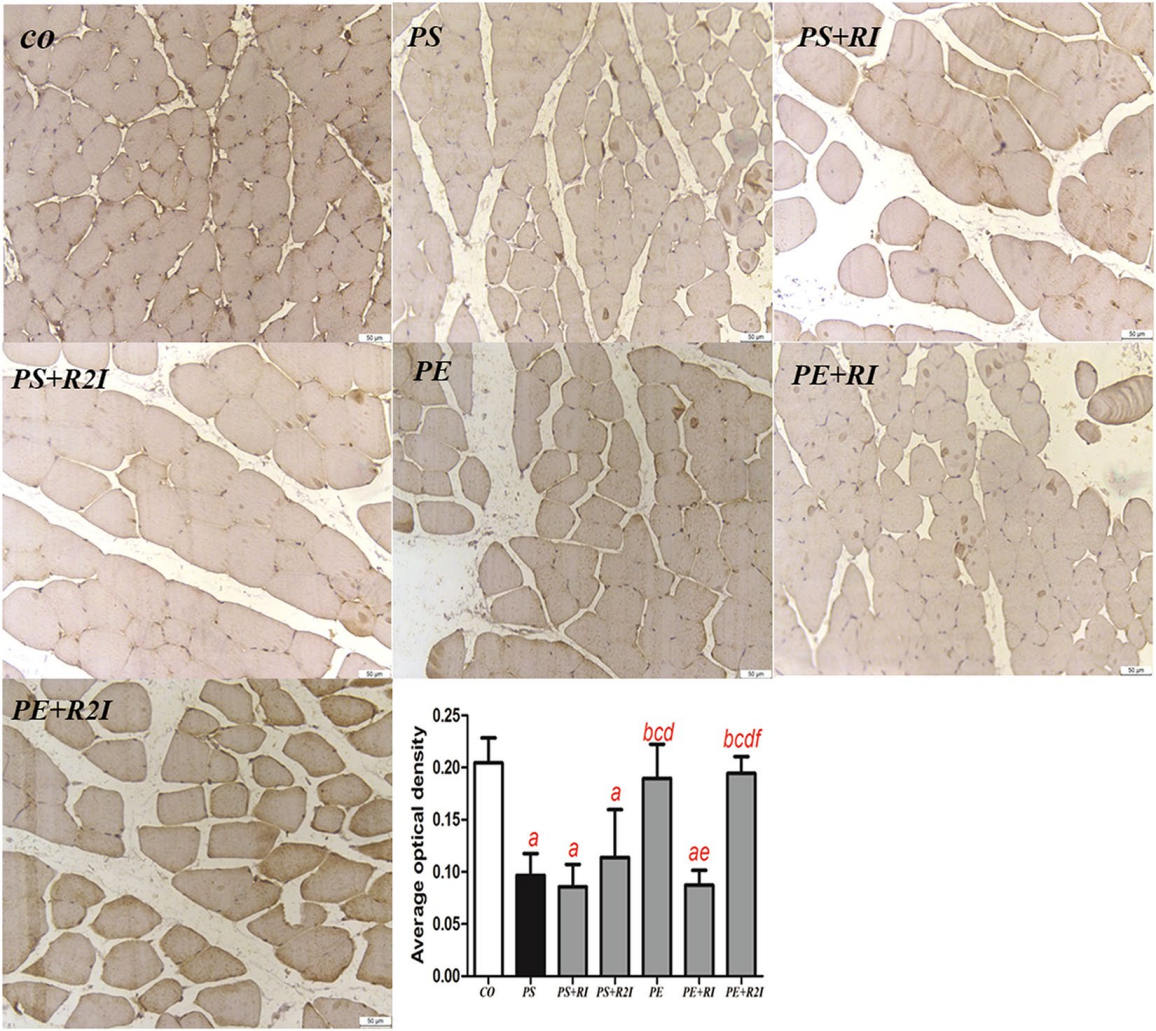
Immunohistochemical localization of GLUT4 in skeletal muscle for all groups (×200). ^a^Compared with the CO group, P < 0.05; ^b^compared with the PS group, P < 0.05; ^c^compared with the PS + RI group, P < 0.05; ^d^compared with the PS + R2I group, P < 0.05; ^f^compared with the PE + RI group, P < 0.05.

Signals were visualized by DAB. Brown staining represents GLUT4 and blue staining represents nuclei. The value of the average optical density of GLUT4 in the CO, PS, PS + RI, PS + R2I, PE, PE + RI and PE + R2I groups were 0.20 ± 0.02, 0.10 ± 0.02, 0.11 ± 0.05, 0.09 ± 0.02, 0.19 ± 0.03, 0.19 ± 0.02 and 0.09 ± 0.02, respectively. The value of the average optical density of GLUT4 in the skeletal muscle of rats in the PS, PS + RI, PS + R2I and PE + RI groups were much less than that of the CO, PE and PE + R2I groups (P < 0.05) (Fig. 7).

## Discussion

Several studies have suggested that insulin resistance plays a key role in the pathogenesis of PCOS^22,23^. By measuring the activity of 5αR in urine, an increase of the 5αR activity was observed with an increase of insulin resistance index in related studies^24–26^. A small sample population-based research study showed that the elevated activity of 5αR was associated with obesity^27^ and type2 diabetes^28^ and could be regulated by the composition of dietary nutrients. Compared with weight-matched normal women, PCOS showed more severe insulin resistance with increased 5αR activity^4^. In our experiment, the PCOS rat model also showed severe insulin resistance with raised activity of 5αR1 in skeletal muscle. Tomlinson *et al*. found that the level of 5αR increased in obese populations, with no gender differences. Therefore, it is suggested that the increase of reductase activity may be a compensatory mechanism after the decrease of insulin sensitivity^29^.

5αRI, which reduce circulating and prostatic 5α-dihydrotestosterone (DHT) levels, are currently widely used in androgen treatment for prostate hyperplasia and other diseases^30–32^. Finasteride(5αR2I) inhibits 5αR2 selectively, whereas dutasteride(5αRI) inhibits both 5αR1 and 5αR2. Previous studies have suggested that there is almost no significant difference between the two inhibitors in terms of clinical efficacy and related parameters^33,34^. However, increasing studies show that the two inhibitors are very different in their biochemical effects^15,35^. Related studies have shown that people who received dutasteride displayed increased body fat and decreased insulin sensitivity. These features were not found in people who received finasteride^35^. In fact, finasteride, in contrast, can even improve insulin function. Therefore, 5αR1 may play an important role in regulating metabolism. In 2014, the study of skeletal muscle cultures of PCOS patients showed no interaction between insulin and androgen^13^. In our present study, PCOS rats showed no improvement in glucose and lipid metabolism after 14 days of intragastric administration of finasteride and dutasteride. Typical characteristics of insulin resistance were observed in these PCOS rats. It was further confirmed that PCOS rats may have abnormalities in some genes or proteins, thus blocking the interaction between androgen and insulin^36^.

Exercise itself reduced 5α-reductase in PCOS rats, and the expression of 5αR1 in skeletal muscle was significantly lower in the PCOS exercise group than in the PCOS stationary group. Exercise can reduce body weight and improve insulin sensitivity. Studies have shown that weight loss may be accompanied by a decrease in reductase activity, and the mechanism may be related to the decrease of the glucocorticoid production rate and the role of the hypothalamus pituitary adrenal axis^27^. Similarly, PCOS patients who received an oral insulin sensitizer (thiazolidinediones) showed a decrease in reductase activity. It is considered that the reduction of the 5αR activity was associated with the level of insulin-like growth factor 1(IGF-1), adiponectin, and growth hormone in the circulation^28^. Animal experiments in 2011 suggest that aerobic exercise training induced to increase circulating and muscle DHT level, and the increased muscle DHT level leads to increase in glucose metabolism in skeletal muscle of obese and diabetic rats^37,38^. The results of these studies seem to be in contradiction with the results of reducing the activity of reductase in our experiment. But different from obese and diabetic patients, PCOS patients have the endocrine environment of hyperandrogenism and this study does not pay much attention to androgen pathway. So it remains to be further studied.

It has been recognized and accepted that exercise can improve insulin resistance in diabetes. The main mechanism is to increase the utilization efficiency of insulin by activating the non-insulin dependent AMPK signaling pathway and the insulin-dependent PI3K/AKT signaling pathway^9^. Previous studies have shown that PCOS patients have abnormal glucose metabolism as represented by their levels of AKT. Additionally, animal experiments demonstrated that the changes in glucose metabolism could be induced by exogenous androgen in normal SD rats^11^. Therefore, we chose the PI3K/AKT signaling pathway to investigate. Exercise can improve insulin resistance of PCOS with the main target organ of exercise being skeletal muscle. Therefore, it may be considered that the executive organ of exercise for glucose metabolism regulated by insulin and androgen is skeletal muscle. Insulin is now thought to activate PKC and Akt, as well as AS160, which is a downstream signaling molecule of Akt. By activating PI3K, the sugar transporter GLUT-4 is translocated to the cell membrane and the sugar transport capacity of the muscle cells are all increased^39^, thereby allowing this pathway to be utilized by contracting muscles. Our study has proved that there is an abnormal activation of the PI3K/AKT signaling pathway in the skeletal muscle of the PCOS rat model. After exercise intervention, the PCOS exercise group showed a significantly higher expression of PI3Kp85 protein, as well as the phosphorylation levels of Akt(Ser473) and Akt(Thr308) in comparison to the PCOS stationary group. This suggests that exercise activates the PI3K/AKT signaling pathway in skeletal muscles. In the PCOS non-exercise group, only the reductase inhibitor was given with no increase in the phosphorylation levels of PI3K and AKT being achieved regardless of whether 5αRI or 5αR2I was used. This suggests that the reductase inhibitor itself, although similar to exercise, may decrease reductase activity; however, it cannot activate the PI3K/AKT signaling pathway. In the PCOS exercise group, when type 1 reductase is retained, exercise can activate the signaling pathway by repressing 5αR1 action. These results suggest that 5αR1 may play a key role in the activation of the PI3K/AKT signaling pathway in PCOS rats. Of course, exercises play a comprehensive role and do not exclude the activation of other substances, such as IGF and insulin receptor. Our research suggests that reductase may be another important mediator in addition to known mediators that can regulate PI3K/AKT pathway. And the relationship between these mediators needs further research.

This study confirmed that the expression of 5αR1 protein was increased in skeletal muscle of PCOS rats, and exercise can decrease 5αR1 to activate the PI3K/Akt pathway in skeletal muscle of PCOS rats. Because 5αR1 acts as a key enzyme in the androgen metabolism pathway, this study further confirms that 5αR1 may be at the intersection of two pathways for exercise, androgen therapy and insulin. Thus, the treatment of 5αR1 may be the key to activate the two pathways of androgen and insulin. And, 5αR1, as the target of exercise therapy, maybe can provide a new idea for the formulation of exercise prescription and the evaluation of exercise efficacy in the future. At present, some patients with PCOS exhibit clinical hirsutism and other clinical manifestations of androgen hyperactivity. This group of patients needs to be treated with anti-androgen drugs. This study may put forward a new direction for the clinical use of anti-androgen drugs. That is, anti-PCOS therapy should be performed on the basis of 5αR1 without being blocked, in order to ensure the therapeutic effect on other clinical phenotypes.

There were still some deficiencies in this experiment: (1) In this experiment, we only studied the glucose metabolism pathway and did not assess androgen metabolism in skeletal muscle; (2) The sample size of this study is too small and the study is limited to animal research; (3) In this study, we mainly observed the changes of 5αR1. The relationship between 5αR2 and 5αR1 should be further studied.

## Conclusion

In this study, the PCOS model rats were treated with the combination of 5αR inhibitors and exercise. The results demonstrate that exercise activates glucose metabolism signaling pathways in skeletal muscle by reducing the expression of 5αR1 protein in PCOS rats.

## Electronic supplementary material

supplementary information

## References

[1] Harrison CL, Lombard CB, Moran LJ, Teede HJ (2011). Exercise therapy in polycystic ovary syndrome: a systematic review. Human reproduction update.

[2] Fauser BC (2012). Consensus on women’s health aspects of polycystic ovary syndrome (PCOS): the Amsterdam ESHRE/ASRM-Sponsored 3rd PCOS Consensus Workshop Group. Fertility and sterility.

[3] Moran LJ, Lombard CB, Lim S, Noakes M, Teede HJ (2010). Polycystic ovary syndrome and weight management. Women’s health.

[4] Benrick A (2013). Resveratrol is not as effective as physical exercise for improving reproductive and metabolic functions in rats with dihydrotestosterone-induced polycystic ovary syndrome. Evidence-based complementary and alternative medicine: eCAM.

[5] Wu C, Lin F, Qiu S, Jiang Z (2014). The characterization of obese polycystic ovary syndrome rat model suitable for exercise intervention. PloS one.

[6] Qiu S (2009). Exercise training improved insulin sensitivity and ovarian morphology in rats with polycystic ovary syndrome. Hormone and metabolic research = Hormon- und Stoffwechselforschung=Hormones et metabolisme.

[7] Hoeger KM (2008). Exercise therapy in polycystic ovary syndrome. Seminars in reproductive medicine.

[8] Badawy A, Elnashar A (2011). Treatment options for polycystic ovary syndrome. International journal of women’s health.

[9] Li T (2017). Role of the PI3K-Akt Signaling Pathway in the Pathogenesis of Polycystic Ovary Syndrome. Reproductive sciences.

[10] Yoshioka M, Boivin A, Ye P, Labrie F, St-Amand J (2006). Effects of dihydrotestosterone on skeletal muscle transcriptome in mice measured by serial analysis of gene expression. Journal of molecular endocrinology.

[11] Sato K, Iemitsu M, Aizawa K, Ajisaka R (2008). Testosterone and DHEA activate the glucose metabolism-related signaling pathway in skeletal muscle. American journal of physiology. Endocrinology and metabolism.

[12] Corbould A (2008). Effects of androgens on insulin action in women: is androgen excess a component of female metabolic syndrome?. Diabetes/metabolism research and reviews.

[13] Eriksen MB (2014). Testosterone treatment increases androgen receptor and aromatase gene expression in myotubes from patients with PCOS and controls, but does not induce insulin resistance. Biochemical and biophysical research communications.

[14] Boda D, Paun D, Diaconeasa A (2009). Evaluation of 5-alpha reductase activity on cultured fibroblast in patients with hyperandrogenemia. Romanian journal of internal medicine = Revue roumaine de medecine interne.

[15] Upreti R (2014). 5alpha-reductase type 1 modulates insulin sensitivity in men. The Journal of clinical endocrinology and metabolism.

[16] Morifuji M, Sanbongi C, Sugiura K (2006). Dietary soya protein intake and exercise training have an additive effect on skeletal muscle fatty acid oxidation enzyme activities and mRNA levels in rats. The British journal of nutrition.

[17] Kumazaki M (2011). Influence of dosing time on the efficacy and safety of finasteride in rats. The Journal of pharmacology and experimental therapeutics.

[18] Xu Y, Dalrymple SL, Becker RE, Denmeade SR, Isaacs JT (2006). Pharmacologic basis for the enhanced efficacy of dutasteride against prostatic cancers. Clinical cancer research: an official journal of the American Association for Cancer Research.

[19] Zhang M, Lv XY, Li J, Xu ZG, Chen L (2008). The characterization of high-fat diet and multiple low-dose streptozotocin induced type 2 diabetes rat model. Experimental diabetes research.

[20] Purkayastha S (2011). Neural dysregulation of peripheral insulin action and blood pressure by brain endoplasmic reticulum stress. Proceedings of the National Academy of Sciences of the United States of America.

[21] Wu C, Wei K, Jiang Z (2017). 5alpha-reductase activity in women with polycystic ovary syndrome: a systematic review and meta-analysis. Reproductive biology and endocrinology: RB&E.

[22] Yue F (2017). [Subclinical hypothyroidism and endocrine metabolic characteristics in women with polycystic ovary syndrome]. *Zhong nan da xue xue bao*. Yi xue ban=Journal of Central South University. Medical sciences.

[23] Cataldo NA (2006). Metabolic and ovarian effects of rosiglitazone treatment for 12 weeks in insulin-resistant women with polycystic ovary syndrome. Human reproduction.

[24] Jakimiuk AJ, Weitsman SR, Magoffin DA (1999). 5alpha-reductase activity in women with polycystic ovary syndrome. The Journal of clinical endocrinology and metabolism.

[25] Tsilchorozidou T, Honour JW, Conway GS (2003). Altered cortisol metabolism in polycystic ovary syndrome: insulin enhances 5alpha-reduction but not the elevated adrenal steroid production rates. The Journal of clinical endocrinology and metabolism.

[26] Blumenfeld Z (2016). Cortisol-Metabolizing Enzymes in Polycystic Ovary Syndrome. *Clinical medicine insights*. Reproductive health.

[27] Tomlinson JW, Finney J, Hughes BA, Hughes SV, Stewart PM (2008). Reduced glucocorticoid production rate, decreased 5alpha-reductase activity, and adipose tissue insulin sensitization after weight loss. Diabetes.

[28] Glintborg D (2009). A randomized placebo-controlled study on the effects of pioglitazone on cortisol metabolism in polycystic ovary syndrome. Fertility and sterility.

[29] Tomlinson JW (2008). Impaired glucose tolerance and insulin resistance are associated with increased adipose 11beta-hydroxysteroid dehydrogenase type 1 expression and elevated hepatic 5alpha-reductase activity. Diabetes.

[30] Andriole GL, Kirby R (2003). Safety and tolerability of the dual 5alpha-reductase inhibitor dutasteride in the treatment of benign prostatic hyperplasia. European urology.

[31] Amory JK (2007). The effect of 5alpha-reductase inhibition with dutasteride and finasteride on semen parameters and serum hormones in healthy men. The Journal of clinical endocrinology and metabolism.

[32] Hong SK (2010). Effect of the dual 5alpha-reductase inhibitor, dutasteride, on serum testosterone and body mass index in men with benign prostatic hyperplasia. BJU international.

[33] Kotelevtsev Y (1997). 11beta-hydroxysteroid dehydrogenase type 1 knockout mice show attenuated glucocorticoid-inducible responses and resist hyperglycemia on obesity or stress. Proceedings of the National Academy of Sciences of the United States of America.

[34] Morton NM (2001). Improved lipid and lipoprotein profile, hepatic insulin sensitivity, and glucose tolerance in 11beta-hydroxysteroid dehydrogenase type 1 null mice. The Journal of biological chemistry.

[35] Dowman JK (2013). Loss of 5alpha-reductase type 1 accelerates the development of hepatic steatosis but protects against hepatocellular carcinoma in male mice. Endocrinology.

[36] Graupp M, Wehr E, Schweighofer N, Pieber TR, Obermayer-Pietsch B (2011). Association of genetic variants in the two isoforms of 5alpha-reductase, SRD5A1 and SRD5A2, in lean patients with polycystic ovary syndrome. European journal of obstetrics, gynecology, and reproductive biology.

[37] Sato K, Iemitsu M, Aizawa K, Mesaki N, Fujita S (2011). Increased muscular dehydroepiandrosterone levels are associated with improved hyperglycemia in obese rats. American journal of physiology. Endocrinology and metabolism.

[38] Sato K, Nishijima T, Yokokawa T, Fujita S (2017). Acute bout of exercise induced prolonged muscle glucose transporter-4 translocation and delayed counter-regulatory hormone response in type 1 diabetes. PloS one.

[39] Makker A, Goel MM, Das V, Agarwal A (2012). PI3K-Akt-mTOR and MAPK signaling pathways in polycystic ovarian syndrome, uterine leiomyomas and endometriosis: an update. Gynecological endocrinology: the official journal of the International Society of Gynecological Endocrinology.

