# Supplementary material for: Exercise activates the PI3K-AKT signal pathway by decreasing the expression of 5α-reductase type 1 in PCOS rats
Source: Sci Rep. 2018 May 22;8:7982. doi: 10.1038/s41598-018-26210-0 (PMC5964186; doi:10.1038/s41598-018-26210-0)

SREP-18-05580

Exercise activates the PI3K-AKT signal pathway by decreasing the expression of 5α-reductase type 1 in PCOS rats

Chuyan Wu, Feng Jiang, Ke Wei, Zhongli Jiang

Fig3-5αR1

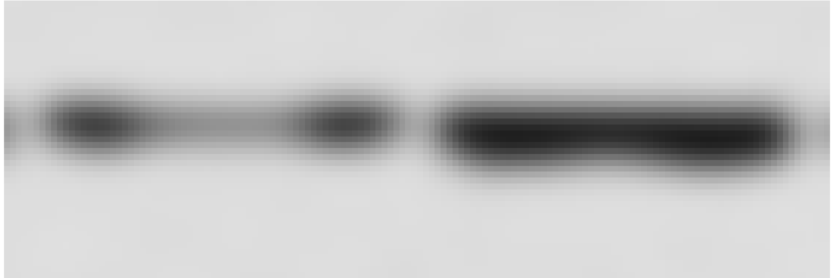

Fig3-5αR1-β-actin

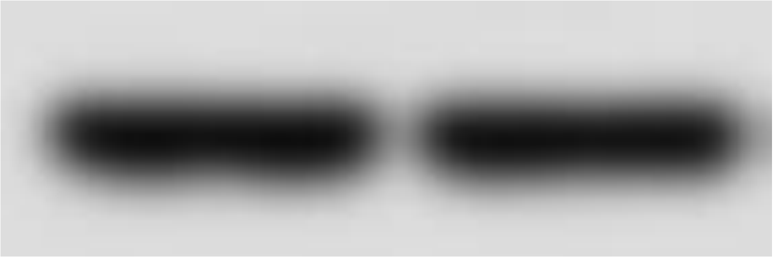

Fig4-5αR1

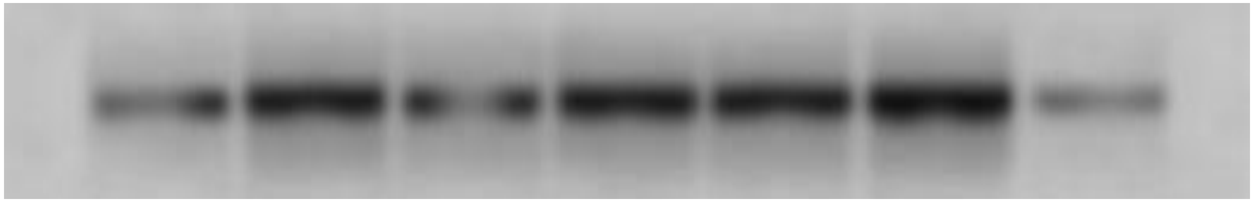

Fig4-β-actin

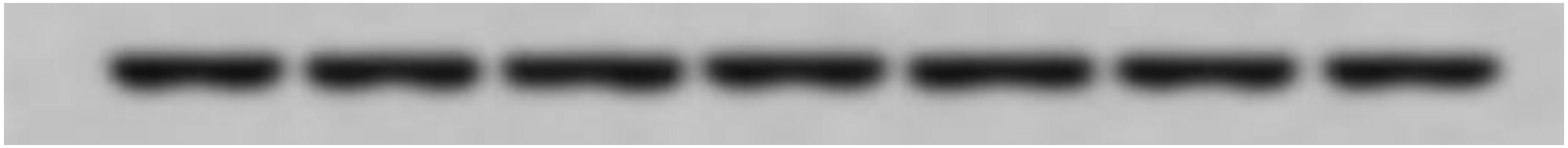

Fig5-Akt Ser 473

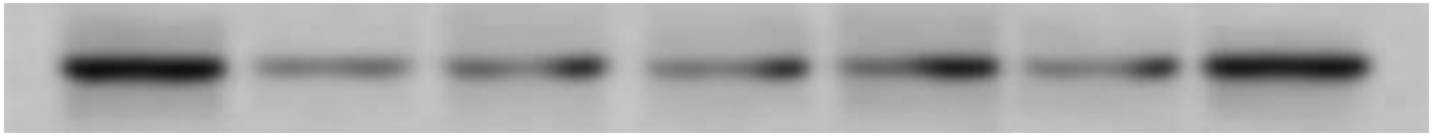

Fig5-Akt Thr 308

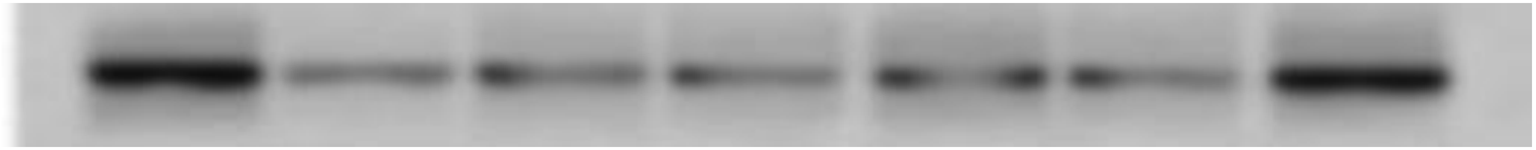

Fig5-PI3K p85

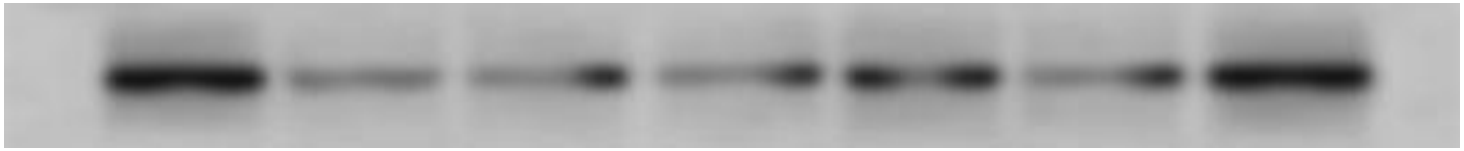

Fig5-β-actin

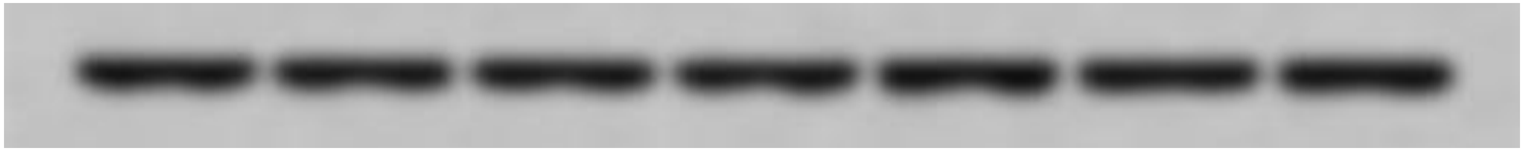

Fig6-PM GLUT4

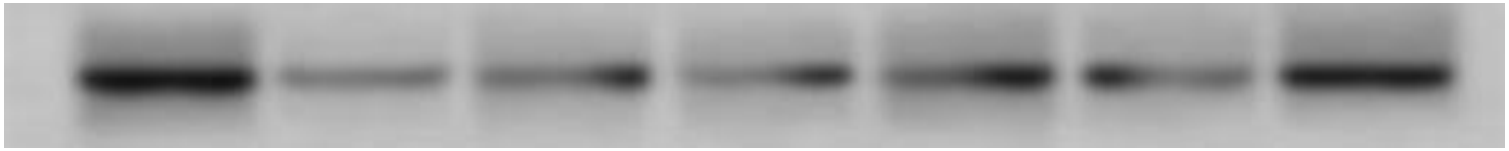

Fig6-Total GLUT4

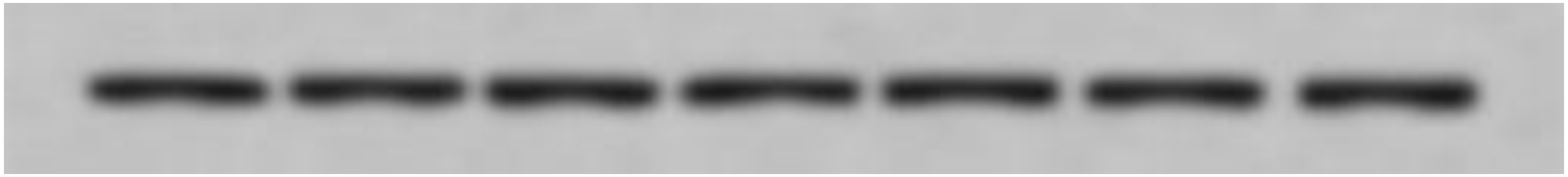

Fig6-β-actin

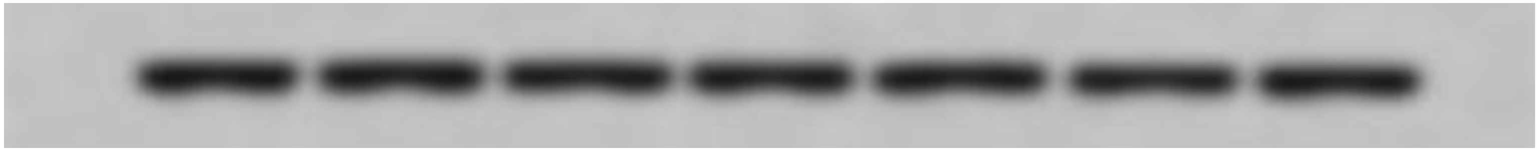

Supplement: Supplementary file 1 — supplementary information [file 41598_2018_26210_MOESM1_ESM.pdf]
